# Experimental studies from shake flasks to 3 L stirred tank bioreactor of nutrients and oxygen supply conditions to improve the growth of the avian cell line DuckCelt®-T17

**DOI:** 10.1186/s13036-023-00349-5

**Published:** 2023-04-24

**Authors:** Valentine Tingaud, Claire Bordes, Eyad Al Mouazen, Claudia Cogné, Marie-Alexandrine Bolzinger, Philippe Lawton

**Affiliations:** 1 LAGEPP, Laboratoire d’Automatique, de Génie des Procédés et de Génie Pharmaceutique, GePharm Team, Université Claude Bernard Lyon 1, CNRS UMR5007, 43 Boulevard du 11 Novembre 1918, Villeurbanne CEDEX, 69622 France; 2 Laboratoire d’Automatique, de Génie des Procédés et de Génie Pharmaceutique, Université Claude Bernard Lyon 1, ISPB, 8 avenue Rockefeller, Lyon, 69373, CEDEX 08 France

**Keywords:** Cell growth, DuckCelt®-T17 avian cell line, Fed-batch culture, Glutamax, Nutrient supplementation

## Abstract

**Background:**

To produce viral vaccines, avian cell lines are interesting alternatives to replace the egg-derived processes for viruses that do not grow well on mammalian cells. The avian suspension cell line DuckCelt^®^-T17 was previously studied and investigated to produce a live attenuated metapneumovirus (hMPV)/respiratory syncytial virus (RSV) and influenza virus vaccines. However, a better understanding of its culture process is necessary for an efficient production of viral particles in bioreactors.

**Results:**

The growth and metabolic requirements of the avian cell line DuckCelt^®^-T17 were investigated to improve its cultivation parameters. Several nutrient supplementation strategies were studied in shake flasks highlighting the interest of *(i)* replacing L-glutamine by glutamax as main nutrient or *(ii)* adding these two nutrients in the serum-free growth medium in a fed-batch strategy. The scale-up in a 3 L bioreactor was successful for these types of strategies confirming their efficiencies in improving the cells’ growth and viability. Moreover, a perfusion feasibility test allowed to achieve up to ~ 3 times the maximum number of viable cells obtained with the batch or fed-batch strategies. Finally, a strong oxygen supply – 50% dO_2_ – had a deleterious effect on DuckCelt^®^-T17 viability, certainly because of the greater hydrodynamic stress imposed.

**Conclusions:**

The culture process using glutamax supplementation with a batch or a fed-batch strategy was successfully scaled-up to 3 L bioreactor. In addition, perfusion appeared as a very promising culture process for subsequent continuous virus harvesting.

**Supplementary Information:**

The online version contains supplementary material available at 10.1186/s13036-023-00349-5.

## Introduction

Embryonated eggs are widely used for the production of viral vaccines since this system allows post-translational modifications and high productivity [1]. Industrially, this method offers the advantage of a fully validated process performed in compliance with global pharmaceutical standards (cGMP). However, the presence of residual contaminants causing many allergic reactions and the longer process time should be in favor of the use of cell culture systems [2]. The strong needs for vaccines against seasonal epidemics or new viruses are pushing research to focus on alternative production systems that are just as safe, robust and, above all, faster and more profitable than the traditional method. The use of mammalian continuous cell lines has many advantages in terms of ethics but also of production yields [3]. Viral vaccine production in cell culture depends on the type of cells used and the type of vaccine desired. In the case of influenza vaccines, the Madin-Darby Canine Kidney (MDCK) cell line has become a reference [4] and produce viral titers equivalent to eggs [5]. The first seasonal influenza vaccine Flucelvax tetra® produced on MDCK cells received a European marketing authorization issued in 2018. Moreover, this line is adherent (as opposed to suspension culture), i.e. it requires a physical support to be able to divide, which complicates the process, especially during the scaling up. Another cell line that may have a real potential for large scale influenza vaccines production is the human embryonic retinal cell (PER.C6) line cultivated in suspension [6]. The PER.C6 cell line provides a robust host system that can replace embryonated eggs for the production of influenza virus vaccines [7]. In addition, a number of avian cell lines is increasingly used to replace the egg-derived processes or for those viruses that do not grow well on mammalian cells such as the highly attenuated modified vaccinia Ankara vaccine (MVA) [8]. Three avian cell lines are competing with traditional mammalian cell platforms used for influenza vaccine productions [9]. The proprietary duck embryonic stem cell line EB66 [10–12] as well as other avian suspension duck retina AGE.CR [8, 13–18] and BA3 ciPC are at an advanced stage of commercial development for the manufacture of biological vaccines. Suspension cell lines present many advantages for the industrialization of a vaccine production process: easy up-scalability, growth in serum-free media, easy handling as well as growth potential at high density [19].

In this context, the avian suspension cell line DuckCelt®-T17 was previously studied and investigated for the production of a live attenuated metapneumovirus (hMPV)/respiratory syncytial virus (RSV) and influenza virus vaccines [20–22]. Infection of DuckCelt®-T17 cells with the vaccine candidate Metavac® was initiated as soon as 1 × 10^6^ cells/mL were reached with a cell viability around 90% to allow efficient viral replication. Upon infection, there was a loss of about 20% in the number of cells and a decrease in their viability [22]. Although the production of influenza particles was reported to be efficient in 400 mL bioreactors [20], hMPV production in bioreactors remained difficult. A thorough understanding of the DuckCelt®-T17 culture process is thus essential since at present the production systems in use are either TubeSpin50 or shake flasks [22].

Cells use the canonical metabolic pathways, the Krebs cycle and glutaminolysis, so they necessarily need carbon sources such as glucose or glutamine to multiply. A better understanding of cell growth is thus required to scale-up the culture process and eventually optimize the production of viral particles. Hence, the purpose of this study was to improve and control the growth kinetics and the metabolic requirements of this cell line both at lab and at pilot scales. For this, we looked at the dO_2_ parameter in the 3 L bioreactor system and at various supplementation strategies in both shake flasks and 3 L bioreactor.

## Materials and methods

### Materials

The OptiPRO™ SFM serum-free medium with penicillin/streptomycin and the surfactant Pluronic F68 was the culture medium. L-glutamine and the L-alanyl-L-glutamine dipeptide (GlutaMAX™ and hereinafter referred to as glutamax) were used as additional nutrients. All these reagents were purchased from Gibco/Thermo Fisher Scientific (Illkirch-Graffenstaden, France).

### Cell culture

The DuckCelt®-T17 cell line was grown in suspension in OptiPRO™ SFM medium supplemented with 1% (v/v) penicillin/streptomycin (10,000 U/mL), 2% (v/v) L-glutamine and 0.2% (v/v) Pluronic F68 as previously described [20]. For lab-scale experiments and routine culture, the cells were cultured at 37 °C in a CO_2_ Kühner LT-XC shaker incubator (Kühner, Birsfelden, Switzerland) with 5% CO_2_ and 85% humidity. After thawing, the cells were cultured in TubeSpin® tubes (TPP® Techno Plastic Products, Trasadingen, Switzerland) at 175 rpm at a working volume of 10 mL for the first passages. Cell amplification was subsequently performed in Erlenmeyer unbaffled shake flasks (DuoCAP®, TriForest, Irvine, CA, USA) at 115 rpm under volumes ranging from 20 to 200 mL until enough cells was obtained for the production phase in bioreactor. Cells were passaged every 2 to 3 days at cell concentrations of 0.7 × 10^6^ cells//mL. The cell passage number was kept between 17 and 30.

### Supplementation studies

The supplementation studies (see Table 1) were performed in 125 mL shake flasks with aerated caps in a working volume of 30 mL inoculated at 0.7 × 10^6^ viable cells/mL and the cultures were carried on for 11 days. Samples were taken daily for growth, metabolic consumption (glucose and glutamine) and production (lactate and ammonium). All supplementations were made with fresh OptiPRO™ SFM medium supplemented with glutamine, surfactant and antibiotics as described above.

**Table 1:** Description of the different supplementation strategies studied at lab-scale in shake flasks with the corresponding DuckCelt®-T17 growth kinetic parameters. For each strategy, the supplemented basic culture medium is the OptiPRO™ SFM medium with antibiotics and Pluronic F68

Strategy	Description	µ_max_ (h^− 1^)	t_D_ (h)	IVCC^a^ (10^6^ cells.d/mL)
**A**	Glutamine (4 mM, D0)	0.023	30.7	26.1 ± 1.1
**B**	Glutamax (4 mM, D0)	0.019	35.7	30.0 ± 1.5 (*)
**C**	Without glutamine	–	–	3.5 ± 0.2
**D**	Glutamine (4 mM, D0) + Glutamax (4 mM, D0)	0.016	43.1	23.0 ± 1.1
**E**	Glutamax (4 mM, D0) + Glutamax (4 mM, D0)	0.019	35.9	29.6 ± 1.1 (*)
**F**	Glutamine (4 mM, D0) + Glucose (D3, D6)	0.019	36.1	29.8 ± 0.7 (*)
**G**	Glutamax (D0) + Glucose (D3, D6)	0.018	39.2	27.8 ± 0.7
**H**	Glutamine (4 mM, D0) + Glutamax (4 mM, D3)	0.018	39.4	26.6 ± 0.7
**I**	Glutamine (2mM, D0) + Glutamine (2mM, D3)	0.019	37.3	31.4 ± 2.2 (*)
**J**	Glutamax (2mM, D0) + Glutamax (2mM, D3)	0.019	37.3	31.0 ± 1.7 (*)
**K**	Glutamine (4 mM, D0) + SFM (D3, D6, D9)	0.019	35.9	31.5 ± 0.4 (*)
**L**	Glutamine (4 mM, D0) + SFM w/o Gln (D3, D6, D9)	0.019	35.4	29.0 ± 0.6 (*)
**M**	Glutamax (4 mM, D0) + SFM (D3, D6, D9)	0.016	43.9	27.0 ± 0.7

^a^ Results are presented as mean ± SEM (n = 6), asterisk (*) indicates a *p-*value < 0.05 in t-test analysis

### Bioreactor assays

The cells were cultured in an Applikon 3 L glass bioreactor equipped with a marine impeller of the elephant ears type and connected to an eZ-control device (Getinge, Göteborg, Sweden). The working volume was 1.610 L. Oxygen supply was performed by pulsed aeration with air at 300 mL/min when the dissolved oxygen (dO_2_) in the bioreactor fell below the given setpoint (10, 30 or 50%), with a 100% value corresponding to O_2_ saturation concentration in the culture medium in equilibrium with the gas phase (ambient air). The operating parameters were stirring speed (100 rpm), pH (7.2) and temperature (37 °C). The pH was controlled by CO_2_ injection or sodium bicarbonate addition.

Perfusion cell culture was achieved with the implementation of the XCell™ Lab System (Repligen,

Waltham, USA). Cell retention in the bioreactor was done by alternating tangential flow filtration (ATF 2 System) using hollow fiber-based membrane filters (0.2 μm pore size, 0.13 m² surface area). Fresh medium was added, and waste products and depleted medium were continuously removed via peristaltic pumps. The perfusion process was initiated on day 3 when the glucose concentration fell close to the limit concentration of 1 g/L (reference conditions, see part 3.1). Fresh medium was provided at the same rate that product and depleted medium were removed from the bioreactor to maintain a constant working volume. The flow rate was manually increased over the process time from 0.8 mL/min at day 3 to 1 mL/min from day 5 until the end of the culture; the other parameters of the ATF 2 controller were kept as given by the supplier.

### Culture process monitoring

Cell growth monitoring was performed by cell counting and viability evaluation with trypan blue with an Automated TC20 Counter (Bio-Rad. Hercules, USA). Trapezoidal integration of the viable cell concentration over time using GraphPad Prism Software (San Diego, USA) led to the integral of viable cell concentration (IVCC). The growth kinetics parameters *µ*max and t_D_ were graphically determined. Metabolite analyses were performed off-line with a Nova Bioprofile Flex1 Analyzer (Nova biomedical, Waltham, USA) in 1mL samples. To obtain information on the cellular metabolism, consumptions of glutamine and glucose, as well as productions of lactate and ammonium were monitored. Means comparisons were done using Student’s *t*-test with the two-sided level of significance, (α = 0.05).

## Results

### Preliminary experiment in a 3 L bioreactor

**Fig. 1:**
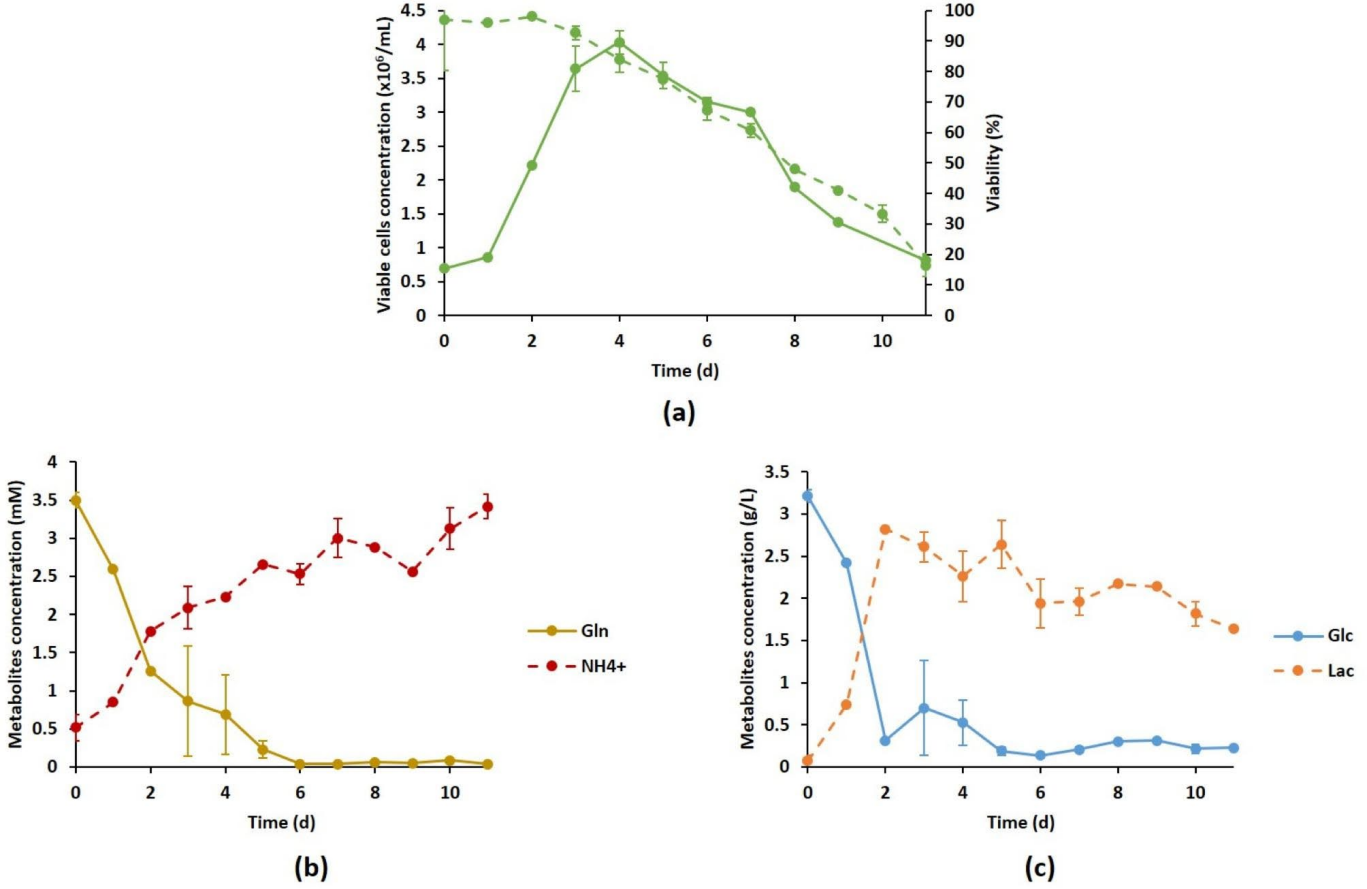
Characterization of the DuckCelt®-T17 culture process performed using reference operating conditions from [20] in a 3 L stirred bioreactor. Time evolution of cell growth (solid line) and viability percentage (dotted line) (a), of the concentration of metabolites involved in glutaminolysis (b) and glycolysis (c). Gln: glutamine (solid line); NH_4_^+^: ammonium (dotted line); Glc: glucose (solid line); Lac: lactate (dotted line). Results are presented as means ± SD (n = 3)

Prior to our investigations, a preliminary experiment was performed to replicate the DuckCelt®-T17 cultivation process as described by Petiot et al. [20] in order to have a reference experiment. Cells were thus cultivated in a 3 L bioreactor with a 50% dO_2_ setpoint and 100 rpm stirring. As shown in Fig. 1a, the growth curve displayed a classical profile, with a maximal viable cell concentration (VCC_max_) of about 4 × 10^6^ cells/mL reached on day 4. The kinetic parameters showed a maximum growth rate (µ_max_) of 0.020 h^− 1^ (± 0.003 h^− 1^) corresponding to a doubling time (t_D_) of 34.7 h (± 0.4 h). The cell viability dropped below 70% from day 5, confirmed by the metabolic kinetic profiles with glucose and glutamine concentrations being close to zero, while the catabolic products lactate and ammonium rose (Fig. 1b and c). The maximal amounts of lactate and ammonium reached during the experiment were 2.8 g/L and 3.5 mM, respectively.

To improve the cultivation process, two strategies were investigated in this study: (i) the modification of the dO_2_ parameter in the 3 L bioreactor system and (ii) the use of different supplementation strategies in both shake flasks and 3 L bioreactor.

### Impact of dO_2_ on the growth and metabolic profiles of DuckCelt^®^-T17 cells in a 3 L bioreactor

**Fig. 2:**
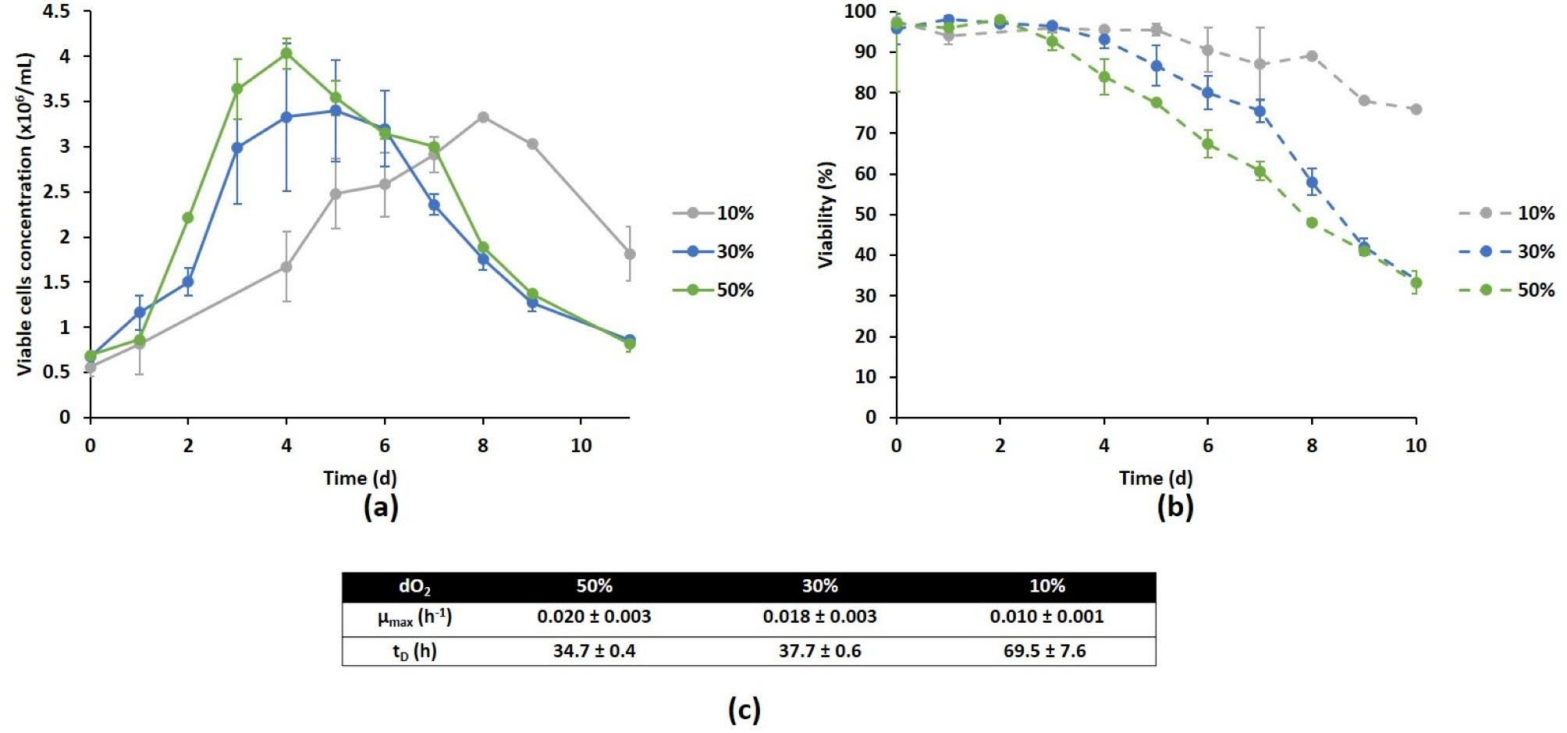
Effect of dO_2_ on cell growth (a), viability (b) and growth kinetic parameters (c) for DuckCelt®-T17 cells cultivated in a 3 L bioreactor. The cells were grown at 10% (grey line), 30% (blue line) or 50% (green line) dO_2_. Results are presented as means ± SD (n = 3 for 30% and n = 2 for 10% dO_2_). The data for 50% dO_2_ (n = 3) originate from the reference process experiments

Since the oxygen needs of the DuckCelt®-T17 line were unknown, the impact of dO_2_ on cell growth was studied at 50, 30 and 10% setpoints with stirring set at 100 rpm (Fig. 2). The growth kinetics were quite similar at 30 and 50% indicating that no O_2_ limitation occurred at 30%. In contrast, the cells seemed to grow significantly slower when the dO_2_ was set at 10%, the peak of cell growth being reached on day 8, compared to days 5 and 6 at 50 and 30%, respectively (Fig. 2a). The maximum growth rate was half as high at 10% than at 30 or 50% (Fig. 2c). Although the VCC_max_ was similar.

whatever the dO_2_ setpoint (about 3.5-4 × 10^6^ viable cells/mL), viability was not. It remained stable until day 3 regardless of dO_2_, but while it dropped drastically at 50% dO_2_, it remained above 70% for a long time at 10% dO_2_ and at 30% dO_2_ it was more stable than at 50% (Fig. 2b).

A clear difference was noticed for metabolic profiles (see Figure S1 in Supplementary Information). For the 10 and 30% dO_2_ setpoints, glutamine was still available at day 7 contrary to 50% (Figures S1a). Ammonium stayed at acceptable levels until days 6–7 (Figures S1b). All these results drove us to set at 30% the dO_2_ parameter as a good compromise for subsequent experiments.

### Study of various supplementation strategies in shake flasks

Given the rapid glucose depletion (see Figure S1c in Supplementary Information) and the high lactate production (data not shown), we especially investigated the effect of glutamax in our system. Indeed, the thermostable dipeptide L-alanine-L-glutamine is cleaved by the proteases produced by the cells, resulting in a sustained release of glutamine in the culture medium. We experimentally observed another advantage of glutamax compared to glutamine: a cell-free process performed for 11 days in the OptiPRO™ SFM medium exhibited spontaneous ammonium production when glutamine was used as a medium supplement while no significant ammonium production was observed with glutamax (see Figure S2 in Supplementary Information).

**Fig. 3:**
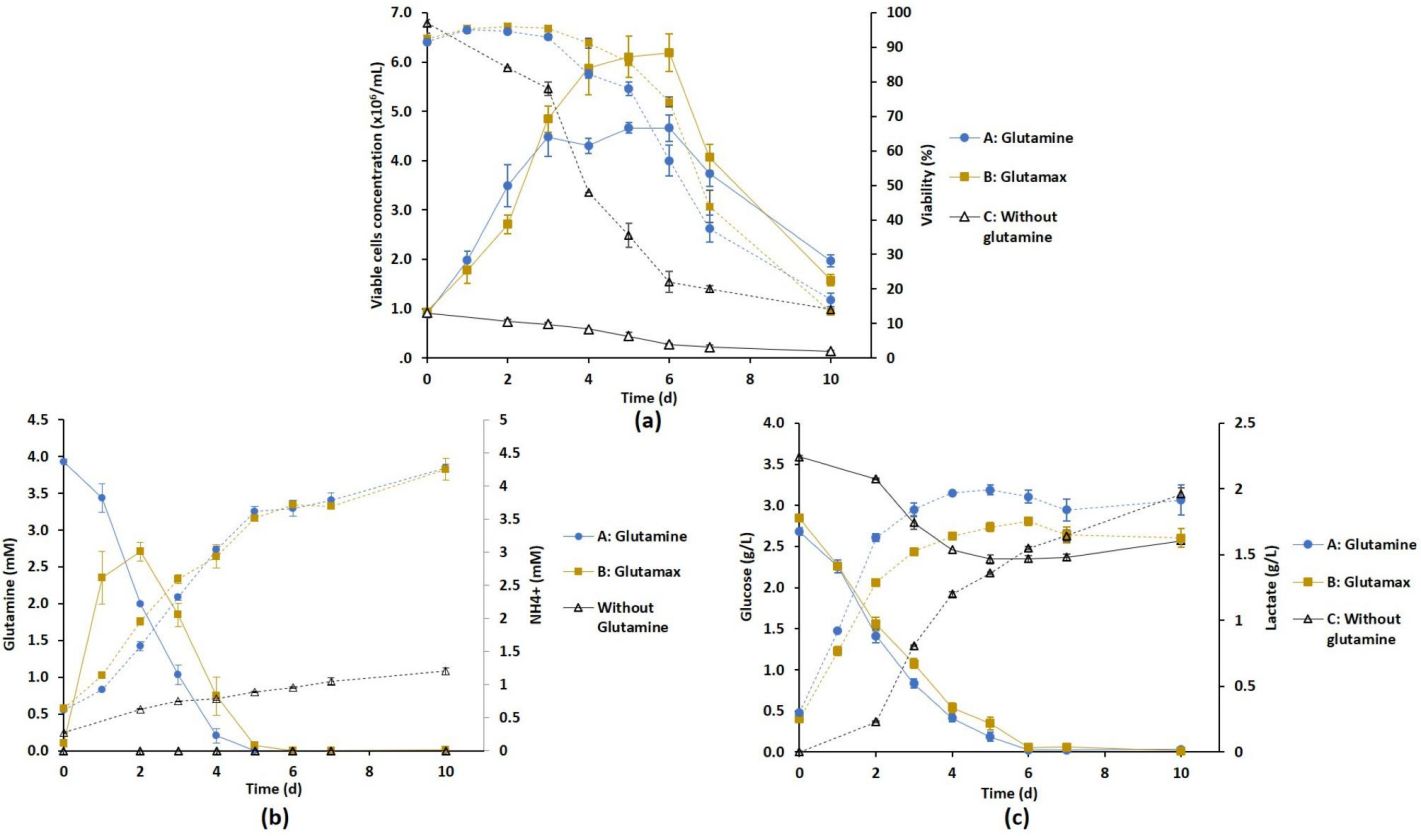
Effect of the substitution of glutamine (strategy A) by glutamax (strategy B) in the culture medium for the DuckCelt®-T17 cell culture in shake flasks. Time evolution of cell growth (solid line) and viability percentage (dotted line) (a), of the concentration of metabolites involved in glutaminolysis (b) and glycolysis (c). Glutamine and glucose are represented in solid line and lactate and ammonium in dotted line. Results are presented as means ± SD (n = 6 for strategies A and B, n = 2 for strategy C (without glutamine))

**Fig. 4:**
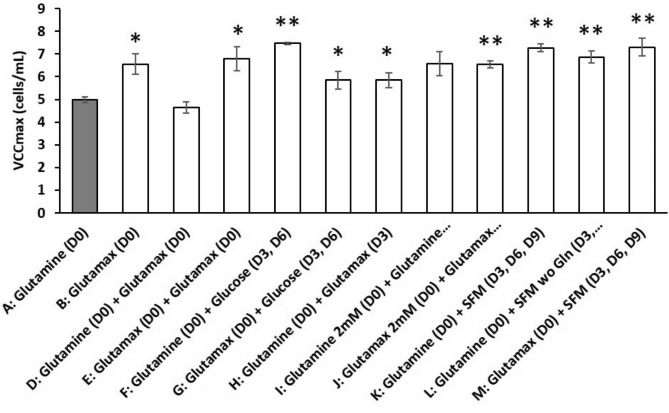
Effect of various supplementation strategies on the maximal concentration of viable cells (VCC_max_) characterizing the DuckCelt®-T17 growth in shake flasks. Results are presented as means ± SD (n = 6 for strategies A, B, D, E and J, n = 3 for the other supplementations conditions). Asterisks (*) and (**) indicate p-value < 0.05 and 0.01, respectively with Student’s t-test

We compared the effect on both cell growth and metabolic profiles of the reference feeding conditions based on glutamine supplementation (strategy A) with various supplementation strategies (Table 1). Strategy B consisted in replacing glutamine by glutamax and as a control, we also performed the cell culture without glutamine (strategy C). The results in terms of cell growth and metabolic profile are shown in Fig. 3. As expected, cells did not grow without glutamine supplementation (strategy C). Strategy B induced the production of greater amounts of glutamine between day 2 and day 5 with a complete depletion observed at day 5 compared to day 4 with strategy A (Fig. 3b). Compared to strategy A, the VCC_max_ was significantly improved with strategy B, since 6.2 × 10^6^ cells/mL were produced on day 5 compared to 4.7 × 10^6^ cells/mL with the reference strategy A (Figs. 3a and 4). IVCC also increased significantly (+ 15% compared to strategy A) (Table 1). However, the kinetic parameters (µ_max_ and t_D_) as well as viability were statistically similar for both conditions (Table 1). Regarding metabolites, glutamax addition had no influence on glucose depletion and ammonium production while interestingly it slightly limited the amount of produced lactate (Fig. 3b and c).

**Fig. 5:**
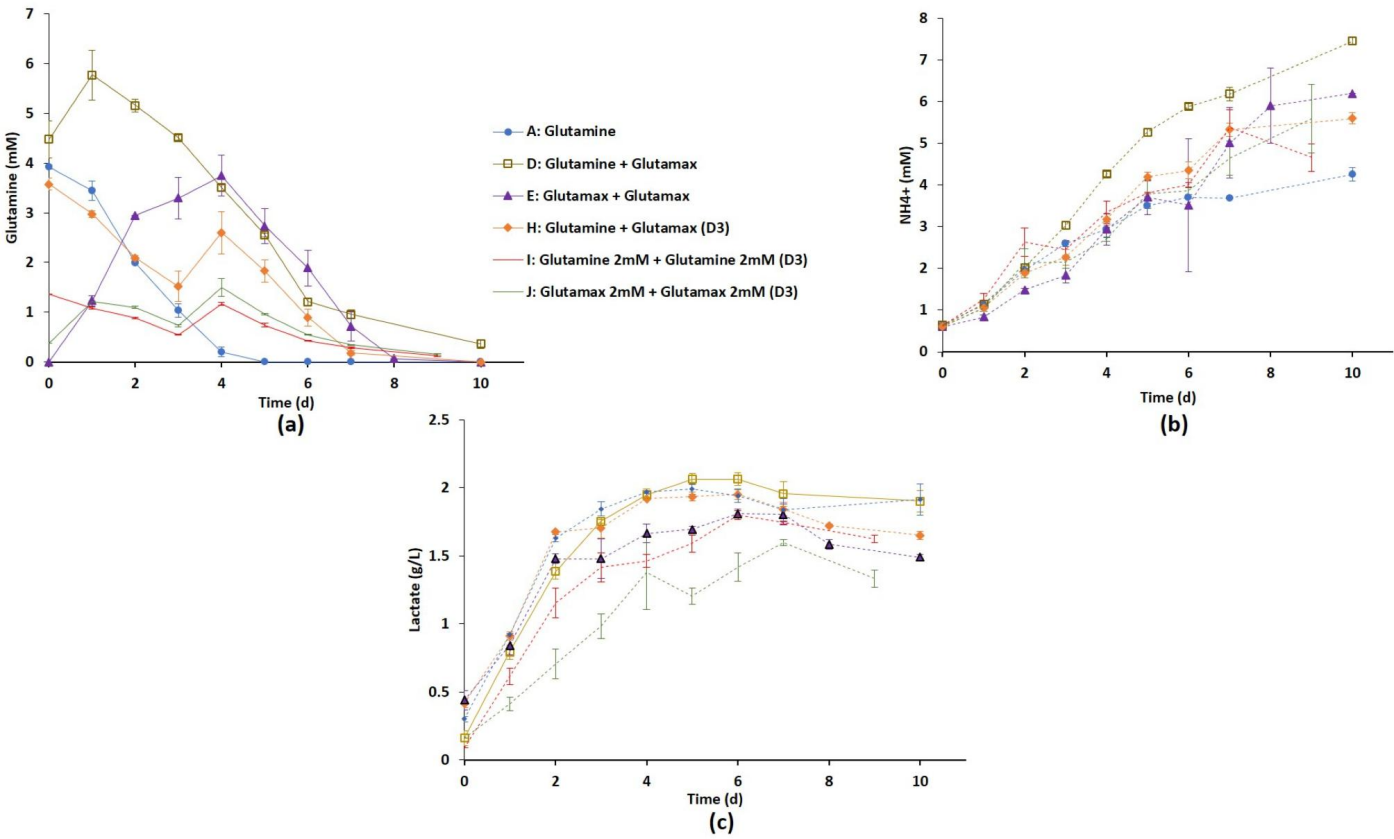
Effect of medium supplementation strategies D, E, H, I and J combining glutamine and/or glutamax as compared to the reference strategy A in shake flasks. Time evolution of glutamine consumption (a) and ammonium (b) and lactate (c) productions during cell culture. Results are presented as means ± SD (n = 6 for strategies A, D, J, n = 5 for strategy E and n = 3 for strategies H and I)

Due to its beneficial effect, glutamax was also added on day 0 (Table 1), either to the reference medium (strategy D) or to the glutamax-supplemented one (strategy E). The combination of glutamine and glutamax had obviously an important influence on the glutamine profile (Fig. 5a). The addition of both nutrients on day 0 led to the highest amounts of glutamine in the medium with ~ 3.5 mM of glutamine still available in the medium at day 4 with a complete depletion only observed on day 7 or 8. But such combinations of nutrients also exhibited the most deleterious effect from an ammonium production point of view (Fig. 5b). Strategy D appeared the most deleterious with a final concentration of ammonium higher than 7 mM inducing low VCC_max_ and IVCC (Fig. 4; Table 1). Although strategy E showed a significant increase of VCC_max_ (6.8 × 10^6^ cells/mL) and IVCC compared to strategy A, it produced greater amounts of ammonium (Figs. 4 and 5b). A similar addition was also tested on day 3 (strategy H) to control the glutamine profile at lower concentration over days and limit the amount of ammonium produced. Although obtaining an intermediate concentration of glutamine in the medium until day 7, no statistically significant difference in both viability and metabolic profiles was observed compared to strategies E and F (Figs. 4 and 5).

To observe the impact of glutamine concentration on cell culture, either 4 mM glutamine or glutamax were added one half on day 0 and the other half on day 3 in strategies I and J, respectively. Glutamine concentration remained between 0.5 and 2 mM until day 7 indicating a lower nutrient consumption by the cells than for strategies A and B (Fig. 5). Considering the metabolic profiles, this limited consumption only slightly decreased the lactate produced, the concentration of the other metabolites remaining comparable to strategies A and B, but such supplementation improved the cell viability and especially IVCC ( ~ + 20% compared to strategy A). The best results were obtained with strategy J that allowed maintaining more than 70% cell viability until day 7 whereas with most strategies it dropped below 70% as early as day 5 (strategies A and D) or 6 (strategies E, H, and I) (see Figures S3 and S4 in Supplementary Information). From these results, we hypothesized that a fed-batch type strategy might be an interesting supplementation strategy.

**Fig. 6:**
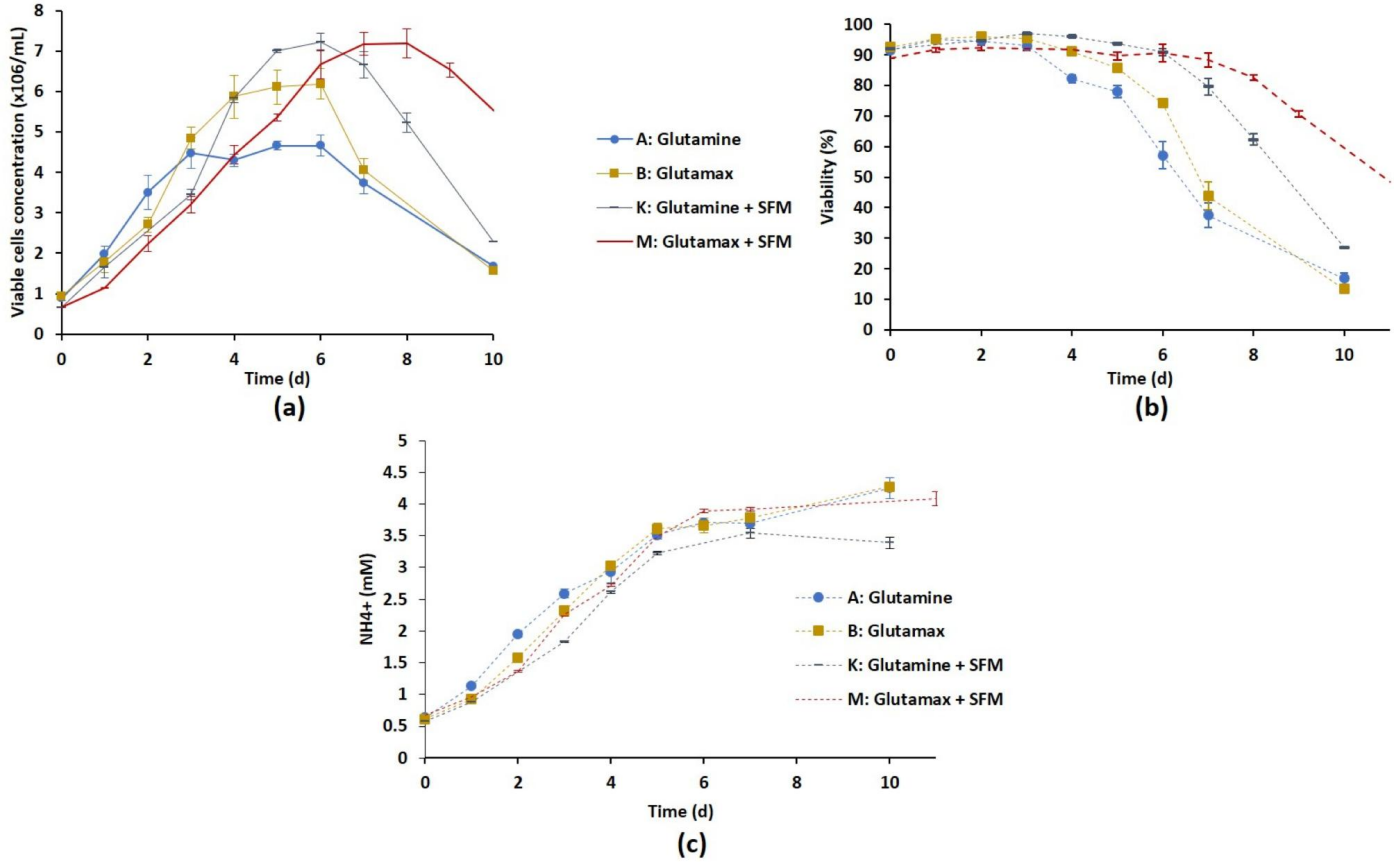
Effect of mimicking fed-batch culture (strategies K and M) by adding OptiPRO™ SFM during the culture in shake flask compared to strategies A and B. Time evolution of cell growth (a), viability percentage (b) and ammonium production (c). Results are presented as means ± SD (n = 6 for strategies A and B, n = 3 for strategies K and M)

Several fed-batch-mimicking strategies were thus investigated. The effect of glucose addition was first studied through strategies F and G that corresponded to A and B, respectively with the addition of 3 g/L glucose on days 3 and 6. Although the VCC_max_ significantly increased (Fig. 4), glucose supplementation during cultivation did not seem to be a good alternative in view of the metabolic profiles observed (see Figure S4 in Supplementary Information). Especially, the lactate concentration sharply increased inducing an acidification of the culture medium. Finally, strategies K and L corresponded to the reference strategy with the addition every 3 days of fresh Optipro SFM medium with or without glutamine, respectively. In strategy M, the initial medium was the glutamax-supplemented one and fresh Optipro SFM with glutamax was added every 3 days. Among these mimicking fed-batch feedings, strategies K and M appeared interesting with VCC_max_ values similar to those of strategy B (Figs. 4 and 6a) and a viability that remained above 70% until 7 and 8 days compared to 5 and 6 days for strategies A and B, respectively (Fig. 6b). Strategy M exhibited slower growth kinetics with lower µ_max_ and higher t_D_ than the other strategies while no significant evolution of IVCC was observed (Fig. 6a; Table 1). Furthermore, the amounts of ammonium and lactate were among the lowest produced as for strategy B (Fig. 6c). Strategies B and M were thus considered as the most promising strategies.

### Scale-up in 3 L-bioreactor and feasibility test

To test the robustness of the DuckCelt®-T17 culture process from shake flask to pilot scale, the cells were cultured in a 3 L bioreactor using the two promising supplementation strategies B and M. Each culture was repeated twice at 100 rpm using 30% dO_2_ and compared to the reference strategy A.

**Fig. 7:**
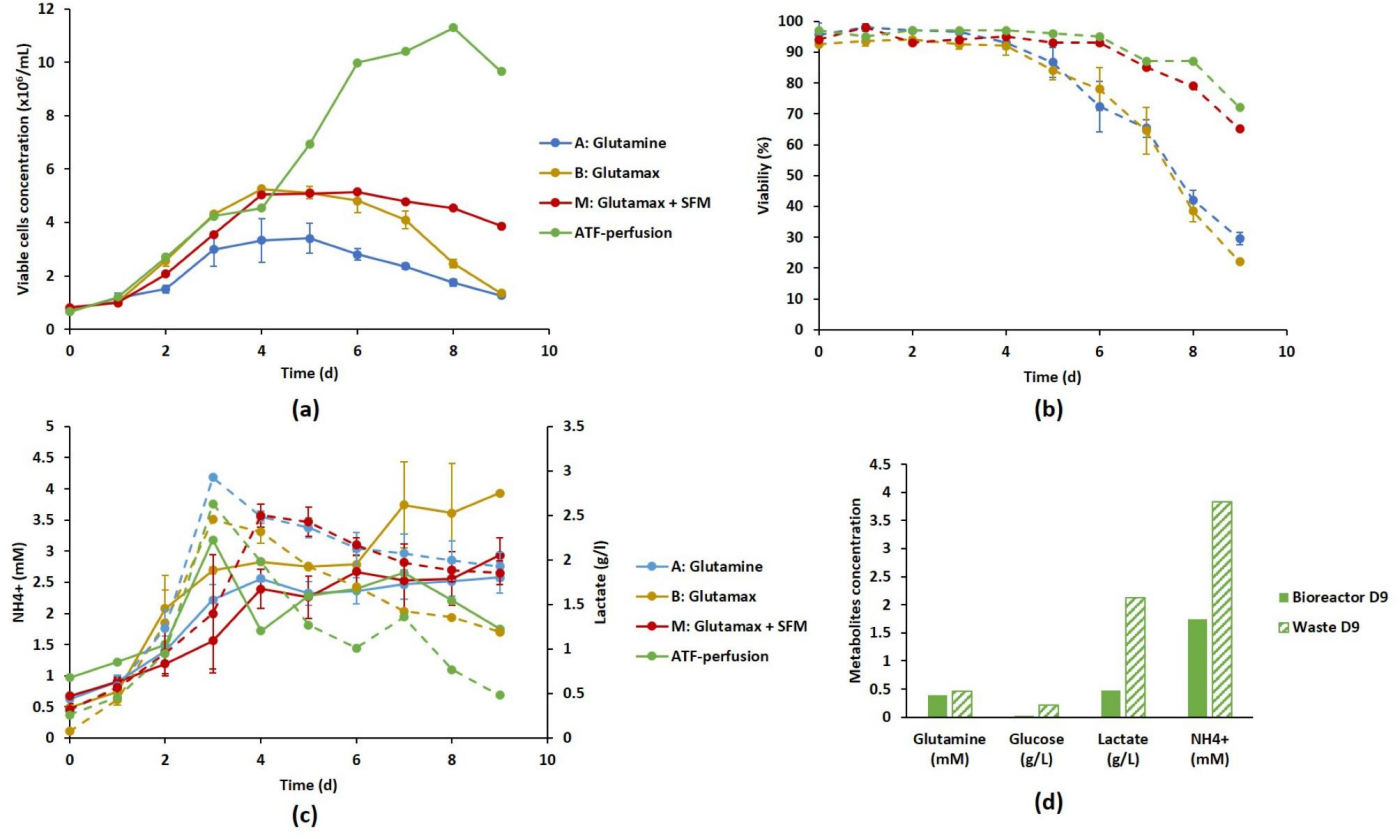
Scale-up in a 3 L bioreactor of the DuckCelt®-T17 culture using batch (glutamine and glutamax), fed-batch (glutamax + SFM) or perfusion processes. Time evolution of cell growth (solid line) (a), viability percentage (dotted line) (b) and waste product concentration (ammonium in solid line and lactate in dotted line) (c). Comparison of metabolic profiles at day 9 between the culture permeate and the bioreactor culture medium (d). Results are presented as means ± SD (n = 3 for strategies A and M, n = 2 for strategy B, n = 1 for perfusion assay)

As observed at lab scale, the substitution of glutamine by glutamax significantly improved the DuckCelt®-T17 cell growth in terms of VCC_max_ (from 3.4 × 10^6^ cells/mL to 5.3 × 10^6^ cells/mL, *p-value* ≤ 0.05) (Fig. 7a). The production of lactate remained below 1.5 g/L compared to 2 g/L for the reference strategy A (Fig. 7c), while no difference in ammonium production and viability profile was observed (Fig. 7b and c). The significant improvement of both cell growth and viability using the fed-batch condition (strategy M) was also observed at pilot scale with a VCC_max_ ~ 5.1 × 10^6^ cells/mL The most promising results in a context of virus production were obtained for the cell viability which remained above 80% during the eight first days of culture against 5 days for strategies A and B (Fig. 7b).

Due to the good results observed with fed-batch cell cultivation, we finally performed a feasibility assay to test the interest of a perfusion strategy. Similar viability profiles were observed with fed-batch and perfusion strategies (Fig. 7b) with a viability maintained above 80% throughout the culture process. But perfusion cell cultivation also greatly improved the cell growth with a VCC_max_ ~1.1 × 10^7^ cells/mL and limited the concentration of lactate and ammonium in the culture medium below 0.5 g/L and 2 mM at the end of the culture, respectively (Fig. 7a, c and d). On the other hand, this process must be optimized since glutamine and glucose (< 0.5 mM and close to 0 g/L respectively) did not remain available to the cells until the end of the culture in the bioreactor despite their continuous addition (Fig. 7d).

## Discussion

As an avian cell line, DuckCelt®-T17 VCC_max_ remains in the low range when compared to the maximum densities reached by AGE1.CR (4–20 × 10^6^ cells/mL) [13] or EB66 cells (8–30 × 10^6^ cells/mL) [10]. Their growth performance, although satisfactory, was slightly lower than some mammalian cell lines classically used for virus production such as Vero (1.0 × 10^7^ cells/mL) [23], MDCK (1.3 × 10^6^ cells/mL) [4] and PER.C6 cells (1 × 10^7^ cells/mL) [6]. After 5 days, their viability loss was confirmed by their metabolic kinetic profiles, consistent with the literature. It is generally agreed that lactate and ammonium should not exceed 1.8 g/L and 2 mM, respectively in the medium to minimize their impact on growth [24] and that the ammonium detrimental effects are often reached at concentrations approximately ten-fold less (e.g., 2–4 mM) than lactate (e.g., 20–40 mM, i.e. 1.8–3.6 g/L) [25]. Thus, decreasing lactate at the expense of even a small increase in ammonium is certainly not desirable.

Oxygen is an essential parameter for aerobic cells’ growth and metabolism [26] and in a bioreactor, dO_2_ is the classical parameter used to control the oxygen supply to the cells. The challenge is to meet the oxygen requirements of the cells without causing cell damage due to bubble burst [27]. Oxygen requirements vary depending on the cell type and the cell line. For example, CHO cells require high levels of oxygen to be very efficient (VCC_max_ around 2.10^7^ cells/mL) and hence dO_2_ is regulated between 30 and 60% with pure oxygen [28, 29]. Avian cells such as the AGE1.CR line are usually grown at 40–55% dO_2_ by pulsed aeration with air enriched with 7.5% CO_2_ and 20% O_2_ [13, 14] or by O_2_ sparging [18]. For the EB66 line, the dO_2_ is set at 50% [10, 11] and for CAP cells, it is around 40–50% by pulsed aeration with pure oxygen [30]. Between 10 and 50% dO_2_, the DuckCelt®-T17 line had similar VCC_max,_ but its viability dropped at 50% dO_2_, possibly due to the more frequent air supply resulting in a greater hydrodynamic stress and cell damage. Such issues could be addressed using pure oxygen or other sparger types, but our configuration did not allow the use of oxygen. The slow cell growth observed at 10% dO_2_ was also associated with better viability, which could be interesting during a virus production phase. But it could be too risky for subsequent virus production if the exponential phase of growth (µ_max_ and t_D_) were not efficient enough to support an infection and if oxygen limitation occurred. In addition, the overproduction of metabolic waste products observed at 10% dO_2_ may have a detrimental influence on viral production performance over time. From an ammonium and lactate production point of view, the intermediate O_2_ supply condition at 30% dO_2_ appeared as a good compromise since ammonium was produced in greatest amounts at 50% dO_2_ and lactate concentrations were well above 1.8 g/L at 10% dO_2_ at the end of the culture.

It is well known that the amounts of nutrients in the medium, essentially glucose and glutamine, are also a limiting criterion for cell growth [31], their rapid depletion leading to a drop in cell viability. In our reference experiment, the glutamine and glucose depletion at day 5 could explain the decline in cell growth. Consequently, we decided to down-scale the culture process using shake flasks, one of the best alternative for carrying out experiments at small-scale level because of their easy operation and lower cost [32]. Due to glutaminolysis and glycolysis associated with the Krebs cycle, the consumption of glucose and glutamine is associated with a high production of lactate and ammonium [24]. Many strategies such as the replacement of glucose with alternative sugars, adapting cells to a lactate-supplemented medium [33], using fed-batch processes [31, 34], were developed to reduce lactate or ammonium accumulation. Moreover, substitution of glutamine with other nutrients such as glutamate is one of the strategies used to reduce the amount of ammonium produced by the cells [35].

To improve the cell growth, we especially investigated the effect of glutamax, since this thermostable dipeptide L-alanine-L-glutamine is cleaved by the proteases produced by the cells, resulting in a sustained release of glutamine in the culture medium. In shake flasks, glutamax allowed increasing growth yields without improving cell viability or metabolic profiles except for lactate production. The same improvements were observed by mimicking a fed-batch process by adding glutamine- or glutamax-supplemented fresh culture medium every three days. Additionally, cell viability was kept above 70% over 7–8 days. When scaling-up to a 3 L bioreactor, the same results were obtained with the fed-batch strategies. The fed-batch condition seems the most promising in view of a virus production.

Overall, DuckCelt®-T17 cells are very sensitive to substrate limitation and inhibited by the waste products, ideally requiring the continuous renewal of the culture medium for both continuous nutriment supply and waste products removal. That is why we performed a feasibility perfusion test, this technique allowing a continuous addition of fresh culture medium across fiber membranes that retain the cells while removing waste products and spent medium. The cell retention is achieved by alternating tangential flow filtration which appears as an process especially adapted to virus production such as influenza A [36]. To our knowledge, this is the first time that such satisfying performances were obtained at this scale with the DuckCelt®-T17 cell line. This is of high interest in a context of virus production.

The DuckCelt®-T17 culture process was successfully scaled-up from shake flasks to a 3 L bioreactor using different supplementation strategies but with a lower culture performance in the latter. These results could be explained by the aeration and hydrodynamic conditions that are not well-controlled in shake flask systems making them difficult to scale-up [27, 32]. The stirring rate as well as the aeration conditions used at pilot scales induce shear stresses that may cause cell damage [37]. Further investigations are required for scale-up purpose especially to deeper characterize the state of the cells and their O_2_ consumption during the culture process and as a function of the operating conditions.

## Conclusions

DuckCelt®-T17 growth and metabolism were investigated using different supplementation strategies in shake flasks. Particularly, the use of glutamax instead of glutamine resulted in a sustained release of glutamine in the culture medium that both favored cell growth and limited the amount of lactate produced. Fed-batch mimicking strategies consisting in adding fresh culture medium every 3 days also improved the DuckCelt®-T17 growth process. At pilot-scale and from an oxygen supply point of view, the best compromise between growth, viability and metabolic profile was obtained at the intermediate dO_2_ of 30%. At 10% dO_2_, the growth kinetics was very slow and the drop in cell viability observed at 50% dO_2_ could be explained by a more frequent air supply that should induce more important cell stress and damage.

The culture process using glutamax supplementation with a batch or a fed-batch strategy was successfully scaled-up to 3 L bioreactor. Finally, perfusion cultivation appeared as a very promising DuckCelt®-T17 culture process since leading to about 1.1 × 10^7^ viable cells/mL as the best VCC_max_ which is very encouraging especially for subsequent continuous virus harvesting. Further experiments may be performed to confirm the reproducibility and to optimize the perfusion process for DuckCelt®-T17 cultivation.

## Electronic supplementary material

Below is the link to the electronic supplementary material.

Supplementary Material 1

Supplementary Material 2

Supplementary Material 3

Supplementary Material 4

Supplementary Material 5

## Data Availability

Please contact author for data requests.

## Abbreviations

dO_2_: dissolved oxygen
Glc: glucose
Gln: glutamine
Lac: lactate
t_D_: doubling time
VCC_max_: maximal viable cell concentration
µ_max_: maximum growth rate

## References

[1] Andleeb R, Ashraf A, Muzammil S, Naz S (2020). Analysis of bioactive composites and antiviral activity of Iresine herbstii extracts against Newcastle disease virus in ovo. Saudi J Biol Sci.

[2] Woodfint RM, Hamlin E, Lee K (2018). Avian Bioreactor Systems: a review. Mol Biotechnol.

[3] Enders JF, Robbins FC, Weller TH (1980). The cultivation of the Poliomyelitis Viruses in tissue culture. Clin Infect Dis.

[4] George M, Farooq M, Dang T, Cortes B (2010). Production of cell culture (MDCK) derived live attenuated influenza vaccine (LAIV) in a fully disposable platform process. Biotechnol Bioeng.

[5] Bardiya N, Bae JH (2005). Influenza vaccines: recent advances in production technologies. Appl Microbiol Biotechnol.

[6] Sanders BP, Edo-Matas D, Custers JHHV, Koldijk MH (2013). PER.C6® cells as a serum-free suspension cell platform for the production of high titer poliovirus: a potential low cost of goods option for world supply of inactivated poliovirus vaccine. Vaccine.

[7] Pau MG, Ophorst C, Koldijk MH, Schouten G (2001). The human cell line PER.C6 provides a new manufacturing system for the production of influenza vaccines. Vaccine.

[8] Jordan I, Vos A, Beilfuß S, Neubert A (2009). An avian cell line designed for production of highly attenuated viruses. Vaccine.

[9] Genzel Y, Reichl U (2009). Continuous cell lines as a production system for influenza vaccines. Expert Rev Vaccines.

[10] Olivier S, Jacoby M, Brillon C, Bouletreau S et al. EB66 cell line, a duck embryonic stem cell-derived substrate for the industrial production of therapeutic monoclonal antibodies with enhanced ADCC activity. *MAbs* 2010, *2*, 405–415. 10.4161/mabs.2.4.12350 PMC3180087 20562528

[11] Léon A, David A-L, Madeline B, Guianvarc’h L (2016). The EB66® cell line as a valuable cell substrate for MVA-based vaccines production. Vaccine.

[12] Nikolay A, Léon A, Schwamborn K, Genzel Y (2018). Process intensification of EB66® cell cultivations leads to high-yield yellow fever and Zika virus production. Appl Microbiol Biotechnol.

[13] Lohr V, Rath A, Genzel Y, Jordan I (2009). New avian suspension cell lines provide production of influenza virus and MVA in serum-free media: studies on growth, metabolism and virus propagation. Vaccine.

[14] Lohr V, Genzel Y, Jordan I, Katinger D (2012). Live attenuated influenza viruses produced in a suspension process with avian AGE1.CR.pIX cells. BMC Biotechnol.

[15] Lohr V, Hädicke O, Genzel Y, Jordan I (2014). The avian cell line AGE1.CR.pIX characterized by metabolic flux analysis. BMC Biotechnol.

[16] Wasik MA, Eichwald L, Genzel Y, Reichl U (2018). Cell culture-based production of defective interfering particles for influenza antiviral therapy. Appl Microbiol Biotechnol.

[17] Aliya Mohamad Ros FN, Abd Rahman N, Ali M, Anuar J (2020). Comparative study between avian cell and mammalian cell in production of Influenza Vaccine Shariah Compliance. IOP Conf Ser Mater Sci Eng.

[18] Coronel J, Gränicher G, Sandig V, Noll T (2020). Application of an inclined settler for Cell Culture-Based influenza a Virus production in Perfusion Mode. Front Bioeng Biotechnol.

[19] Genzel Y (2015). Designing cell lines for viral vaccine production: where do we stand?. Biotechnol J.

[20] Petiot E, Proust A, Traversier A, Durous L et al. Influenza viruses production: Evaluation of a novel avian cell line DuckCelt®-T17. *Vaccine* 2018, *36*, 3101–3111. 10.1016/j.vaccine.2017.03.102 28571695

[21] Lê VB, Dubois J, Couture C, Cavanagh M-H et al. Human metapneumovirus activates NOD-like receptor protein 3 inflammasome via its small hydrophobic protein which plays a detrimental role during infection in mice.PLOS Pathog.2019, 15, e1007689. 10.1371/journal.ppat.1007689 PMC6474638 30964929

[22] Chupin C, Pizzorno A, Traversier A, Brun P (2021). Avian cell line DuckCelt®-T17 is an efficient production system for live-attenuated human metapneumovirus vaccine candidate Metavac®. Vaccines.

[23] Trabelsi K, Majoul S, Rourou S, Kallel H (2014). Process intensification for an enhanced replication of a newly adapted RM-65 sheep pox virus strain in Vero cells grown in stirred bioreactor. Biochem Eng J.

[24] Marc A, Olmos É. Procédés de culture en masse de cellules animales. 2010, 22.

[25] Cruz HJ, Freitas CM, Alves PM, Moreira JL (2000). Effects of ammonia and lactate on growth, metabolism, and productivity of BHK cells. Enzyme Microb Technol.

[26] Martínez-Monge I, Roman R, Comas P, Fontova A (2019). New developments in online OUR monitoring and its application to animal cell cultures. Appl Microbiol Biotechnol.

[27] Nienow AW, Al-Rubeai M (2015). Mass transfer and mixing across the Scales in Animal Cell Culture. Animal Cell Culture.

[28] Nienow AW, Rielly CD, Brosnan K, Bargh N (2013). The physical characterisation of a microscale parallel bioreactor platform with an industrial CHO cell line expressing an IgG4. Biochem Eng J.

[29] Huang Z, Xu J, Yongky A, Morris CS (2020). CHO cell productivity improvement by genome-scale modeling and pathway analysis: application to feed supplements. Biochem Eng J.

[30] Genzel Y, Behrendt I, Rödig J, Rapp E (2013). CAP, a new human suspension cell line for influenza virus production. Appl Microbiol Biotechnol.

[31] Tsao Y-S, Cardoso AG, Condon RGG, Voloch M (2005). Monitoring chinese hamster ovary cell culture by the analysis of glucose and lactate metabolism. J Biotechnol.

[32] Suresh S, Srivastava VC, Mishra IM (2009). Critical analysis of engineering aspects of shaken flask bioreactors. Crit Rev Biotechnol.

[33] Freund N, Croughan M (2018). A simple method to reduce both lactic acid and ammonium production in Industrial Animal Cell Culture. Int J Mol Sci.

[34] Mulukutla BC, Gramer M, Hu W-S (2012). On metabolic shift to lactate consumption in fed-batch culture of mammalian cells. Metab Eng.

[35] Huang H, Yi X, Zhang Y (2006). Improvement of Vero cell growth in glutamate-based culture by supplementing ammoniagenic compounds. Process Biochem.

[36] Hein MD, Chawla A, Cattaneo M, Kupke SY (2021). Cell culture–based production of defective interfering influenza A virus particles in perfusion mode using an alternating tangential flow filtration system. Appl Microbiol Biotechnol.

[37] Grein TA, Loewe D, Dieken H, Weidner T (2019). Aeration and shear stress are critical process parameters for the production of Oncolytic Measles Virus. Front Bioeng Biotechnol.

