# Supplementary figures and images for: Experimental studies from shake flasks to 3 L stirred tank bioreactor of nutrients and oxygen supply conditions to improve the growth of the avian cell line DuckCelt®-T17
Source: J Biol Eng. 2023 Apr 24;17:31. doi: 10.1186/s13036-023-00349-5 (PMC10127095; doi:10.1186/s13036-023-00349-5)

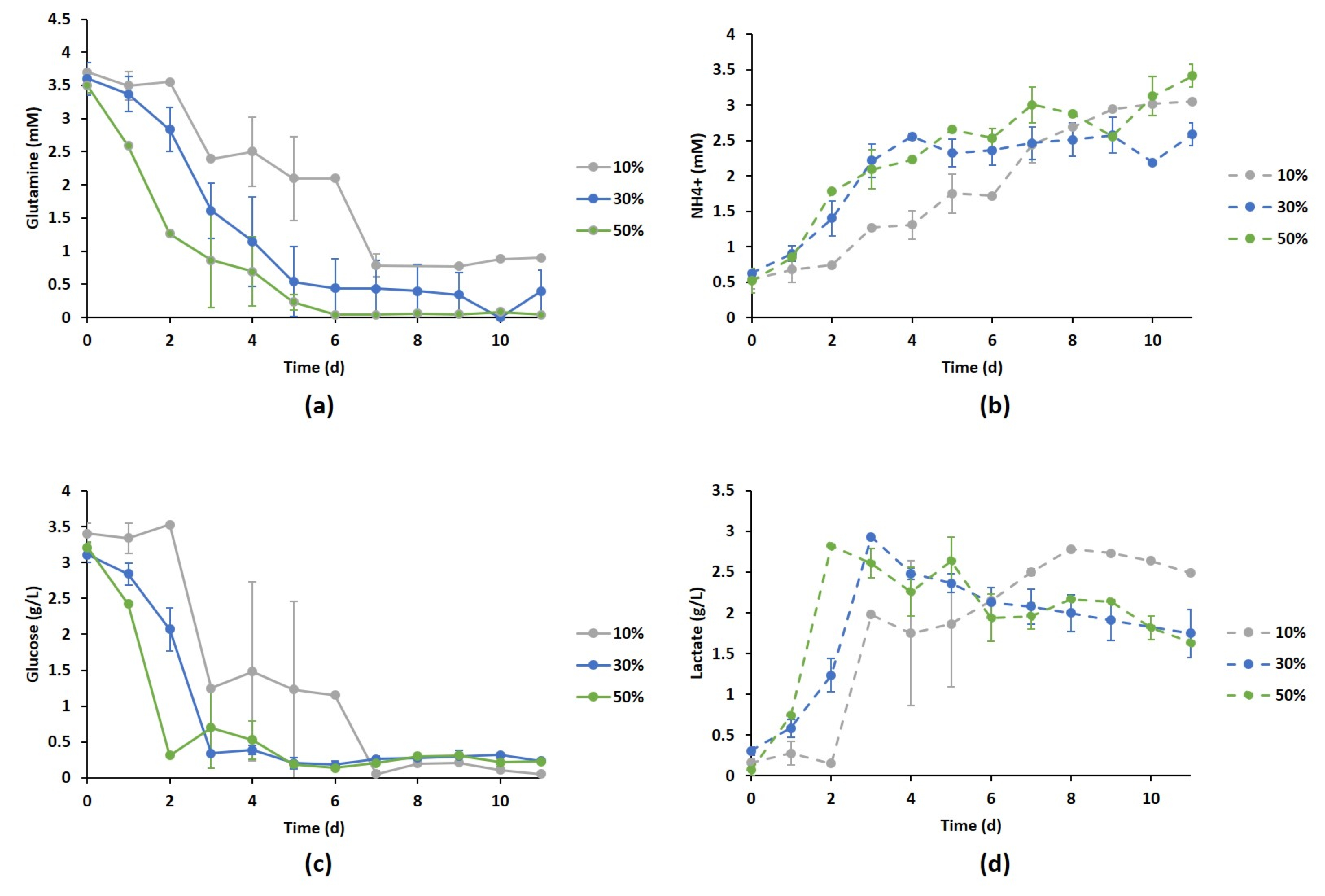

Supplement: Supplementary file 1 — Supplementary Material 1 [file 13036_2023_349_MOESM1_ESM.jpg]

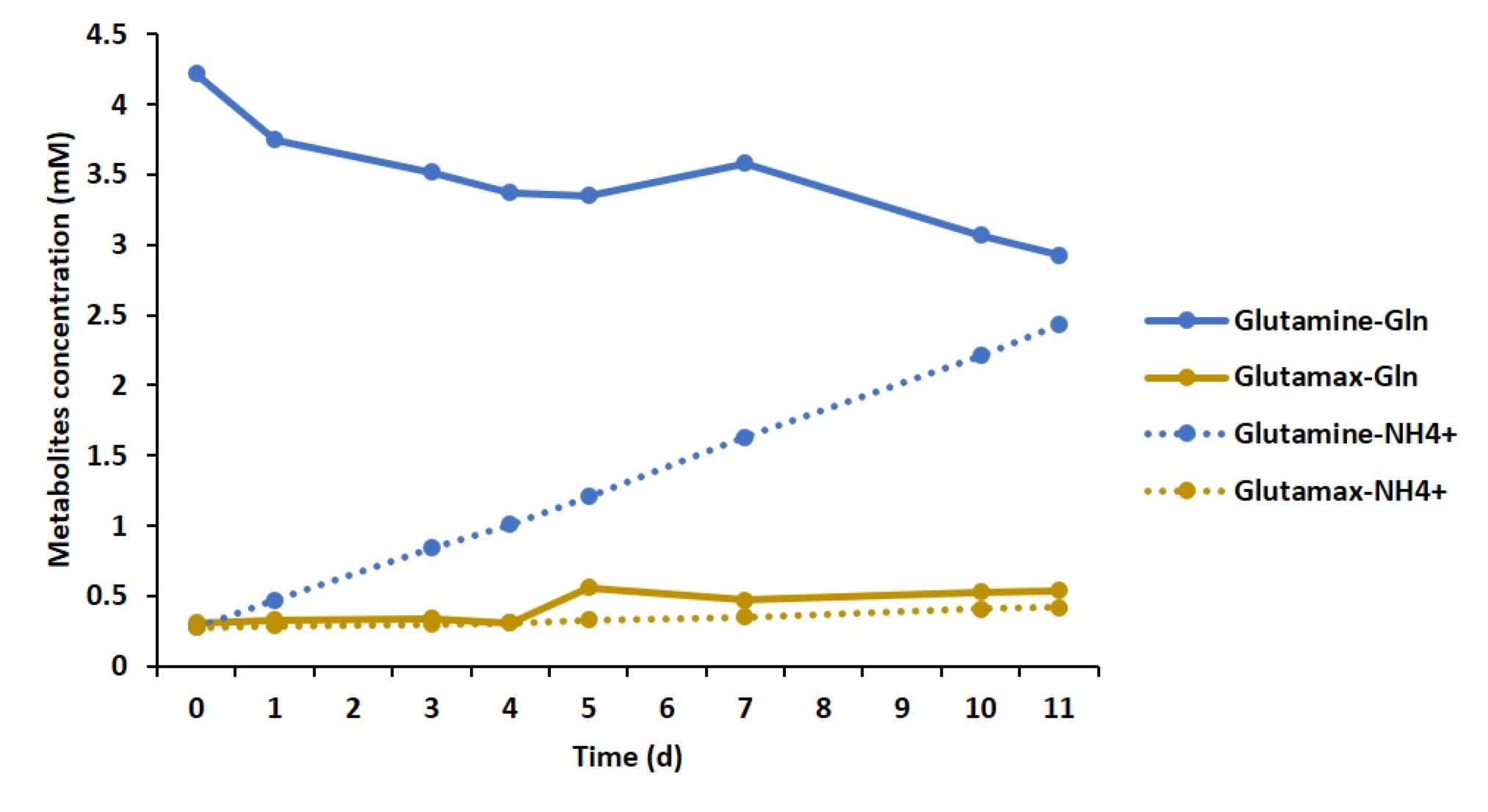

Supplement: Supplementary file 2 — Supplementary Material 2 [file 13036_2023_349_MOESM2_ESM.jpg]

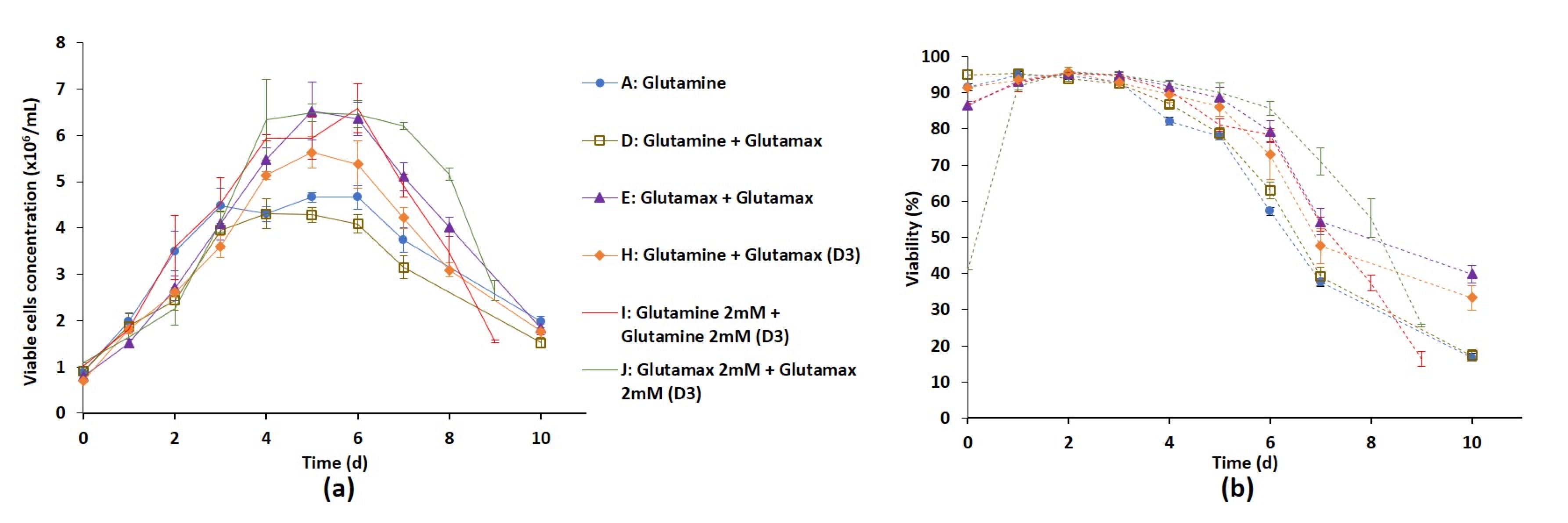

Supplement: Supplementary file 3 — Supplementary Material 3 [file 13036_2023_349_MOESM3_ESM.jpg]

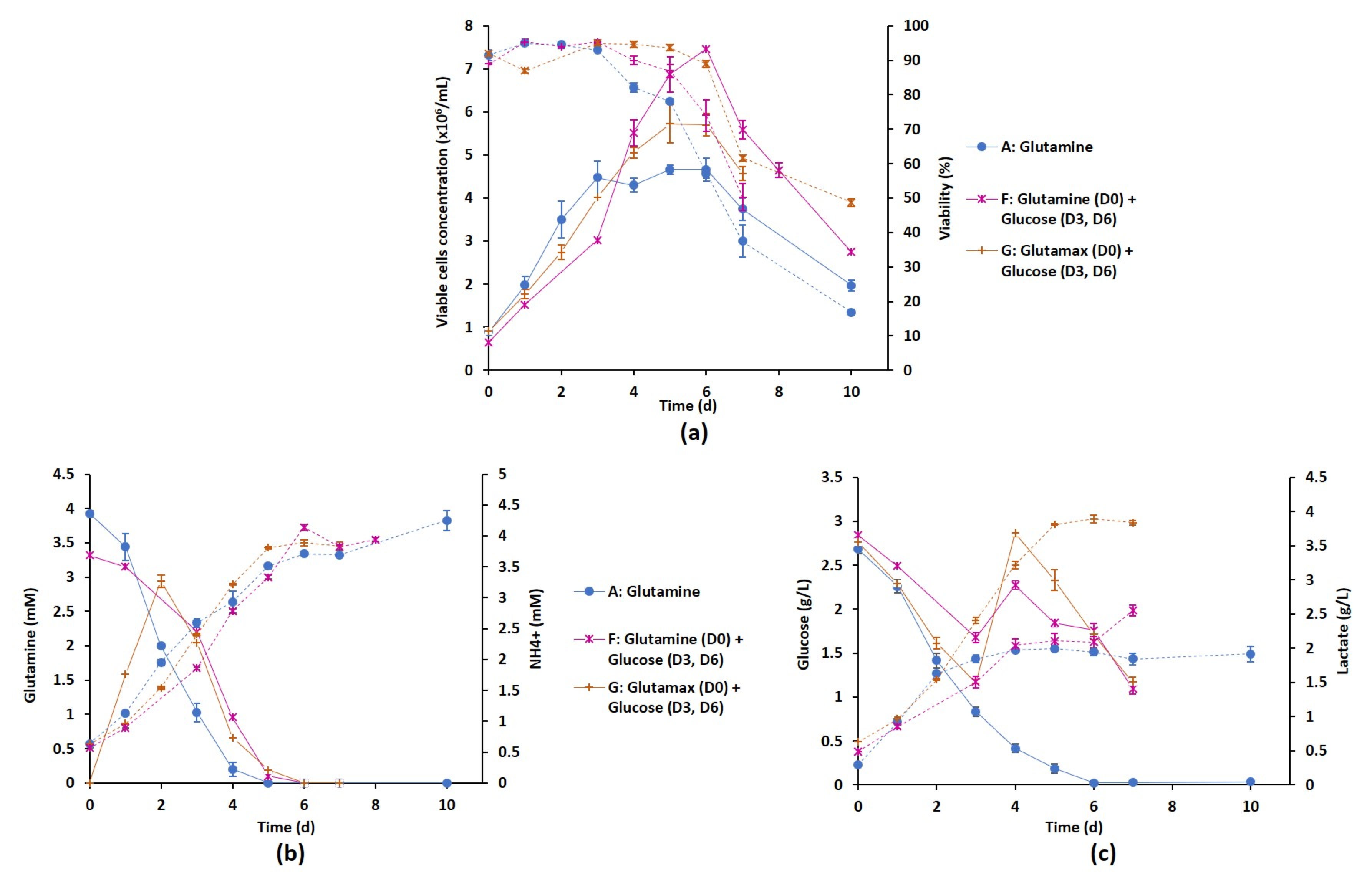

Supplement: Supplementary file 4 — Supplementary Material 4 [file 13036_2023_349_MOESM4_ESM.jpg]
