# Supplementary material for: Experimental studies from shake flasks to 3 L stirred tank bioreactor of nutrients and oxygen supply conditions to improve the growth of the avian cell line DuckCelt®-T17
Source: J Biol Eng. 2023 Apr 24;17:31. doi: 10.1186/s13036-023-00349-5 (PMC10127095; doi:10.1186/s13036-023-00349-5)
Supplement: Supplementary file 5 — Supplementary Material 5 [file 13036_2023_349_MOESM5_ESM.docx]

**Supplementary Information**

**
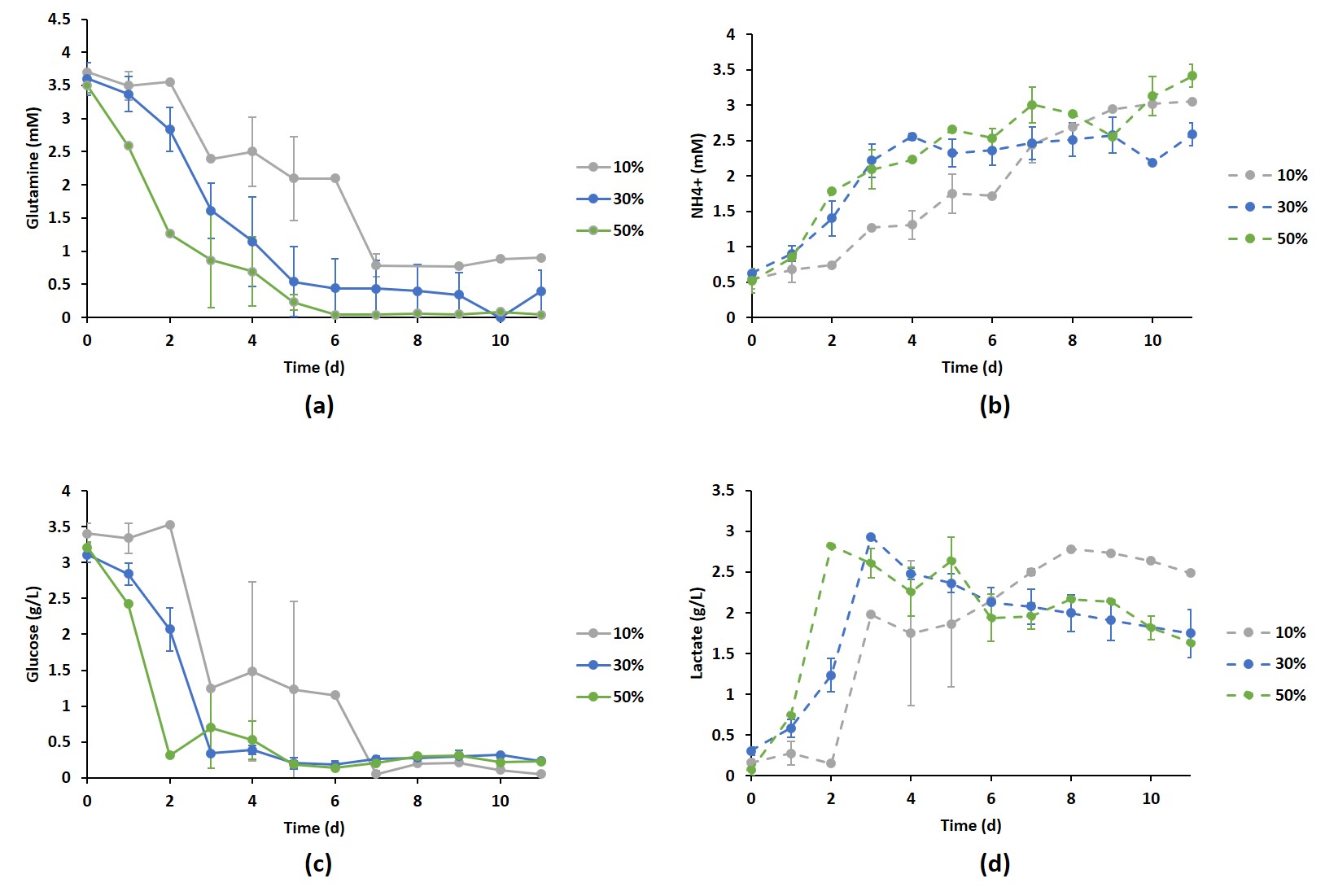
**

**Figure S1:** **Effect of dO_2_ on DuckCelt^®^-T17 cells’ metabolism in a 3L bioreactor.** Time evolution of glutamine (a), ammonium (b), glucose (c) and lactate (d) concentrations in the culture medium. Results are presented as means ± SD (n=3 for 30 and 50 % dO_2_ and n=2 for 10 % dO_2_).

**
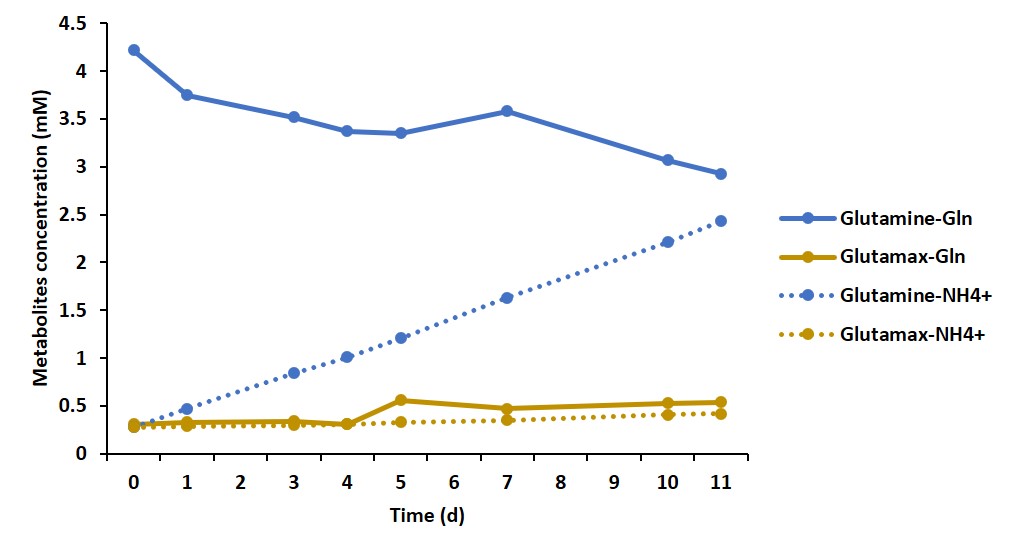
**

**Figure S2: Time evolution in cell-free OptiPRO^TM^ SFM medium of ammonium (dotted line) and glutamine (solid line) concentrations as glutamine (blue) or glutamax (yellow) was used as medium supplement.**

**
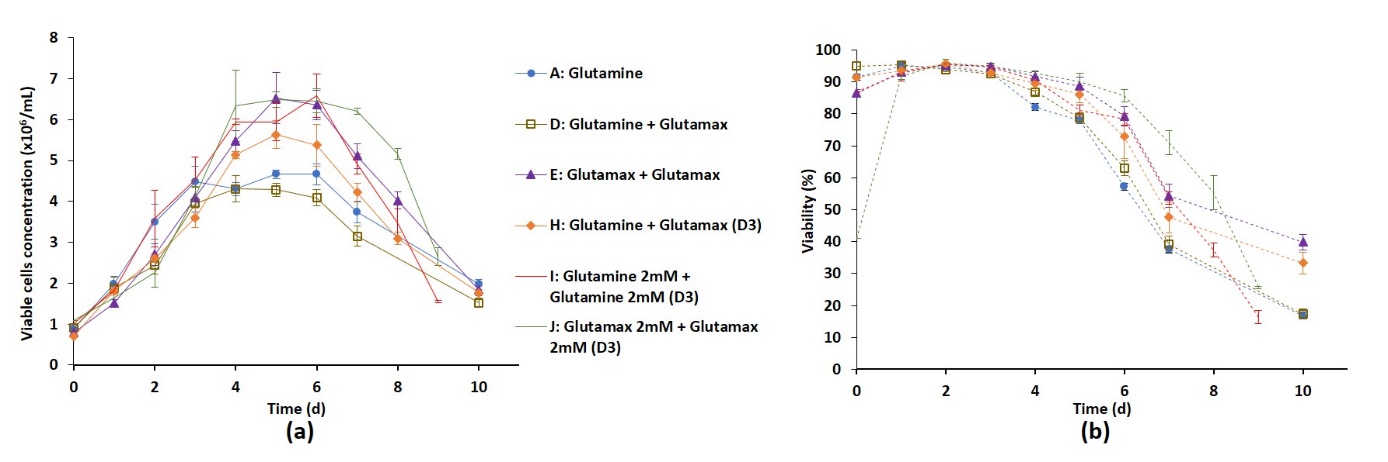
**

**Figure S3: Effect of medium supplementation strategies D, E, H, I and J combining glutamine and/or glutamax as compared to the reference strategy A in shake flasks.** Time evolution of viable cell concentration (a) and viability percentage (b). Results are presented as means ± SD (n=6 for strategies A, D, E and J, n=3 for strategies H and I).


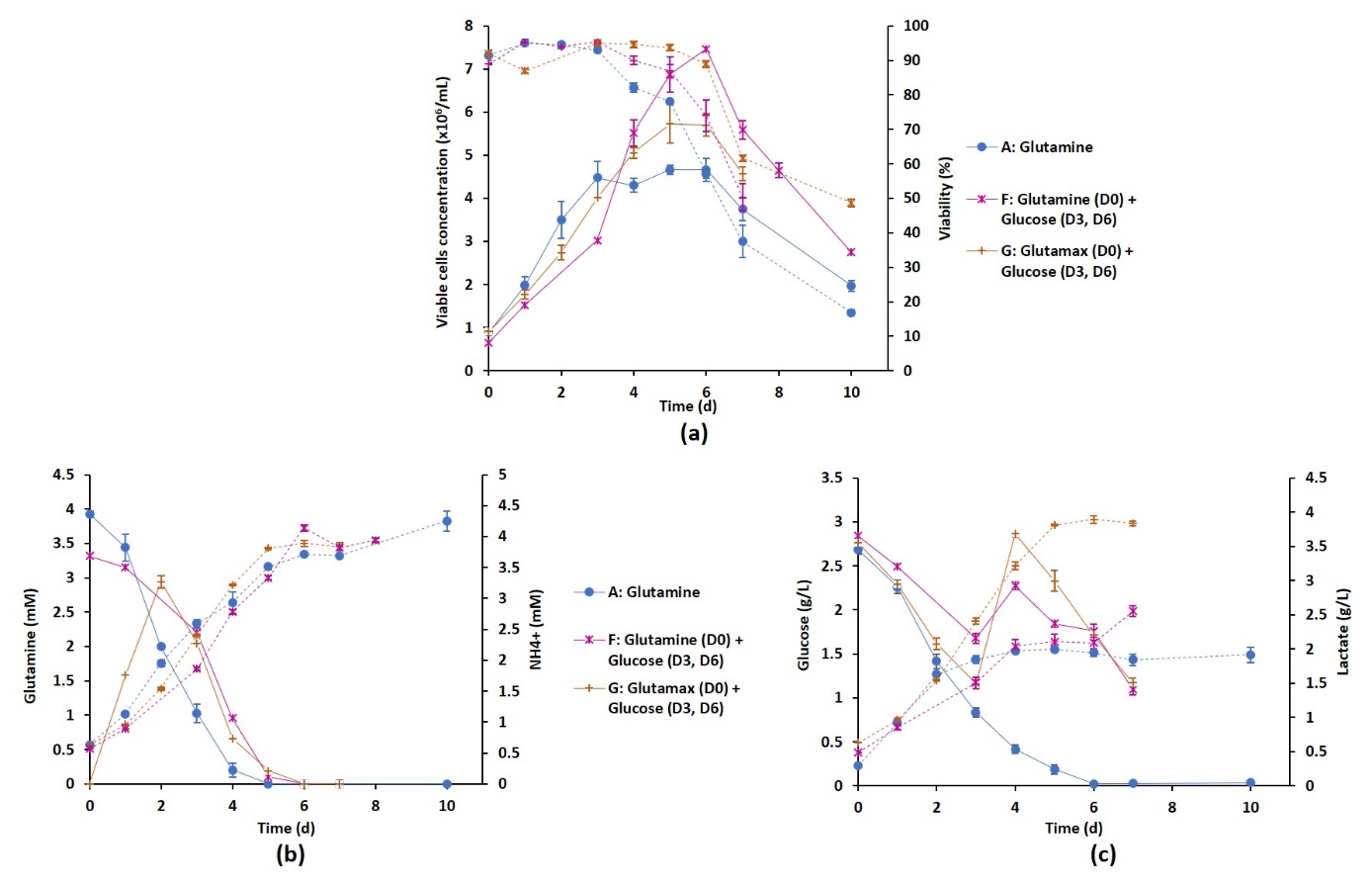


**Figure S4: Effect of the addition of glucose on days 3 and 6 in the OptiPRO^TM^ SFM medium supplemented with glutamine (strategy F) or glutamax (strategy G) in shake flasks.** Time evolution of cell growth (solid line) and viability percentage (dotted line) (a), of cell requirements for metabolites involved in glutaminolysis (b) and glycolysis (c). Glutamine and glucose are represented in solid line and ammonium and lactate in dotted line. Results are presented as means ± SD (n=6 for the reference strategy A, n=3 for strategies F and G).
